# Utility of Saline Infusion Sonohysterography in Gynecology: A Review Article

**DOI:** 10.7759/cureus.35424

**Published:** 2023-02-24

**Authors:** Kingshuk Kumar, Sandhya Pajai, Geeta R Baidya, Krishnendu Majhi

**Affiliations:** 1 Obstetrics and Gynecology, Jawaharlal Nehru Medical College, Datta Meghe Institute of Medical Sciences, Wardha, IND; 2 Obstetrics and Gynecology, Aashirwad Nursing Home, Ghatsila, IND; 3 Medicine, Employees' State Insurance Dispensary, Jugsalai, Jamshedpur, IND

**Keywords:** sis in congenital abnormalities, sis in uterine synechiae, sis in adenomyosis, sis in endometrial carcinoma, sis in submucous fibroid, sis in aub, saline infusion sonohysterosalpingography, saline infusion sonohysterography

## Abstract

This study aimed to describe the role played by saline infusion sonohysterosalpingography (SIS) in the evaluation of uterine lesions. Saline infusion sonohysterosalpingography provides three-dimensional (3D) images with high resolution, which, in turn, gives a good orientation to clinicians and radiologists, in most cases, about the underlying endometrial and tubal pathologies. Saline infusion sonohysterosalpingography is an underused imaging modality that has some advantages over other conventional imaging modalities. It can be used in the diagnosis of gynecological conditions. Saline infusion sonohysterosalpingography gets an edge over other modalities because of its ease of use, cost efficacy, and non-invasive nature while having comparable or even better accuracy than most imaging modalities used in gynecological conditions. Its cost efficacy and excellent pathological characterization make it an imaging modality beneficial for Indian healthcare setups as a whole, and rural healthcare setups in particular where patients cannot afford expensive investigations. This review covers indications and contraindications, imaging technique, drawbacks in imaging, use of SIS in various uterine pathologies, and, in the end, a comparison of SIS with other imaging modalities. Saline infusion sonohysterosalpingography is indicated in most of the prevalent gynecological diseases in India with the reported post-procedural complications being very few. There are a few contraindications as well which should be kept in mind and these are mentioned later. During the procedure, aseptic precautions should be taken. Comparison between imaging modalities will bring out the better modality for a particular case according to the need of the patient.

## Introduction and background

Ultrasonography, also known as ultrasound, is a very widely used non-invasive imaging modality used for imaging the structures underlying skin with almost no contraindications. An ultrasonography machine consists of a transducer connected to a computer screen. The transducer emits waves that reflect back from the underlying structures and are received from the transducer. The information from the reflected waves is interpreted in the computer and is presented as an image on the screen in real-time. It is a very widely used imaging modality for imaging the abdominopelvic organs and other structures lying below the skin in real-time [1]. Saline infusion sonohysterography (SIS), also called saline infusion sonohysterosalpingography, is a radiological imaging modality utilized for imaging various endocervical and endometrial pathologies. Using the same principles as ultrasound, this modality visualizes endometrial cavity which is pre-distended with saline. The best utility of SIS is in detecting and evaluating lesions of endometrial origin and differentiating them from myometrial pathologies. The precision of SIS is at par with hysteroscopy in assessing endometrial lesions [2]. Saline infusion sonohysterography can concurrently evaluate tubal patency with precision at par with hysterosalpingogram (HSG) [3,4].

Saline infusion sonohysterography is performed by injecting sterile saline into the uterine cavity with a catheter for endometrial cavity distension and concurrent pelvic imaging with transabdominal or transvaginal sonography. Aseptic precautions are taken while performing the procedure. These aseptic precautions include using sterile gloves and instruments and cleaning the cervix with cotton swab containing antiseptic solution. Preferably 10-20 mL of warm saline is used for the procedure and is infused slowly. The imaging technique involves the distention of uterine cavity which enables the radiologists or gynecologists to visualize a single layer of uterine cavity. This allows for better characterization of the pathology [5]. Indicated in most gynecological diseases, the only contraindications of SIS are the presence of an active pelvic infection, pregnancy, and presence of an intrauterine device [6]. Saline infusion sonohysterography is a relatively non-invasive procedure, the reported complications are very few, the most common complication being post-procedural infection [7]. Drawbacks of SIS are few, the majority of which can be eliminated by carefully scheduling and performing the procedure. These few drawbacks are easily outweighed by the excellent imaging results of SIS.

Saline infusion sonohysterography is a less used imaging technique, and therefore, the radiologists, clinicians, and trainees are not well acquainted with the technique as well as the imaging. The lack of expertise of a majority of radiologists and the lack of knowledge of superior diagnostic yield of SIS among clinicians is a reason, why it is performed mostly in tertiary care hospitals in India. The most common gynecological diseases along with the conventionally used imaging modality for investigation are described. The results of imaging by SIS are then compared with the conventional imaging method, which in most uterine pathologies is found to be superior. A detailed comparison is drawn between SIS and other imaging modalities, namely transvaginal sonography (TVS), hysteroscopy, hysterosalpingogram, and magnetic resonance imaging (MRI). MRI is the most sensitive imaging modality but cannot be used as a mainstay imaging modality because it is very expensive and time-consuming as well. Imaging modalities have their own set of pros and cons, the comparative study will bring out the best imaging modality for a particular uterine pathology.

## Review

Indications and contraindications

**Figure 1:**
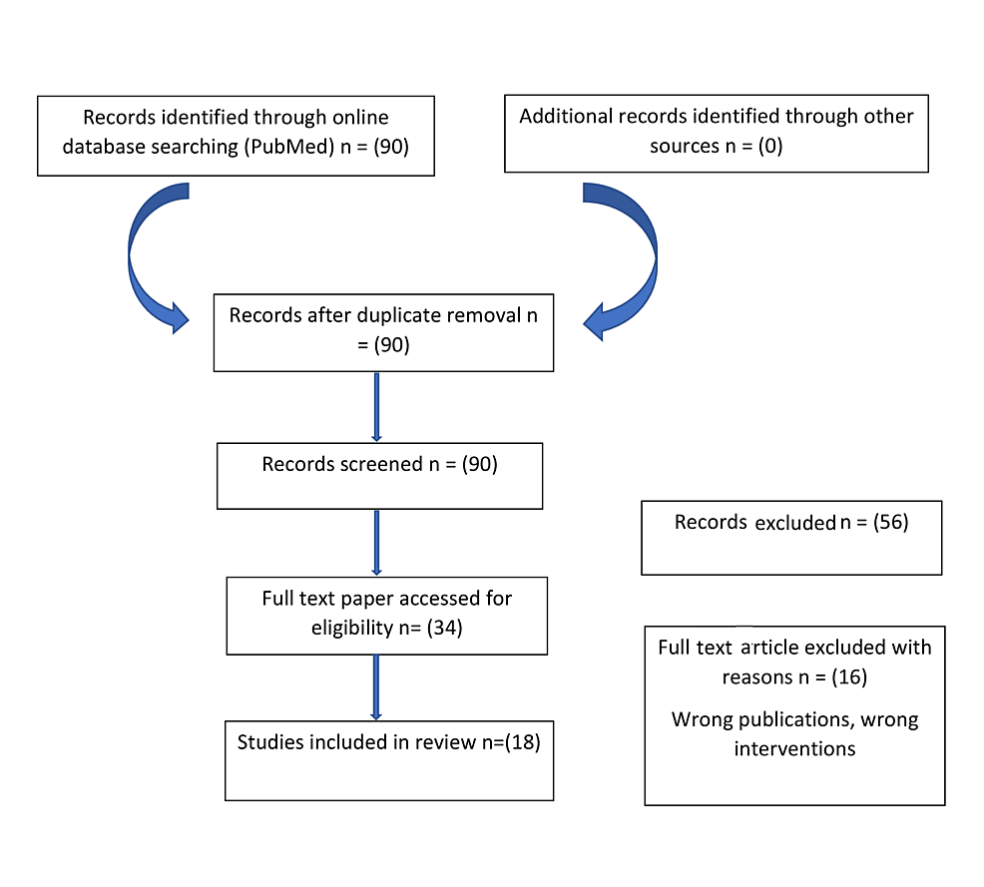
Search methodology used to get the relevant articles.

Abnormal uterine bleeding (AUB) is the most common indication of SIS. AUB is a very prevalent gynecological condition that calls for a complete assessment, especially in the peri-menopausal and post-menopausal age group, as it progresses to endometrial cancer, the incidence being 10-15% in such cases. An endometrial malignancy causing AUB is diagnosed by hysteroscopy-guided endometrial biopsy. Relatively non-invasive techniques like TVS are usually performed as preliminary investigations. However, in screening of endometrial pathologies, the usefulness of TVS is limited [2]. Saline infusion sonohysterography provides better differentiation of focal and diffuse pathologies as it enables the evaluation of a single layer of endometrium by adequate distension of the endometrial cavity during the ultrasound. This permits targeted and precise sampling of focal lesions with superior diagnostic yield. Saline infusion sonohysterography is the second most commonly indicated imaging modality in infertility. Thirty-seven percent of infertility cases are comprised of female factors, while another 35% comprise combined female and male factors. The female infertility factors are categorized as cervical, uterine, ovarian, tubal, and peritoneal [8]. For initial assessment, the primary modality of imaging is TVS. Tubal patency and uterine cavity are evaluated by HSG. Saline infusion sonohysterography is a combination of the features of an HSG and TVS and can simultaneously visualize the tubal patency, uterine cavity, and other pathologies (Figure 1).

Recurrent abortions, pre-operative assessment of submucosal or intracavitary uterine myomas, monitoring after tamoxifen therapy, suspected uterine synechiae, and congenital uterine abnormalities are some other indications for SIS [9,10]. Contraindications for performing SIS are pregnancy, active pelvic infection, and the presence of intrauterine contraceptive devices [6].

Imaging technique

The best time to schedule SIS is between the fifth and 10th days of the menstrual cycle in the early proliferative phase when the endometrial lining is the thinnest [9]. Scheduling in such a way avoids an endometrial polyp or mass being mistaken for a thickened endometrium of the secretory phase which is physiological [11]. The imaging time also prevents saline flushing from dislodging a fertilized ovum during the secretory phase. Due to the presence of residual blood clots in the uterine cavity during the first four days of menstrual cycle, the procedure is avoided as it may be mistaken for pathology. It can be done at any time of the cycle in post-menopausal women suffering from AUB. It is scheduled for patients under hormone replacement therapy at the end of progesterone phase of their menstrual cycle [5].

**Figure 2:**
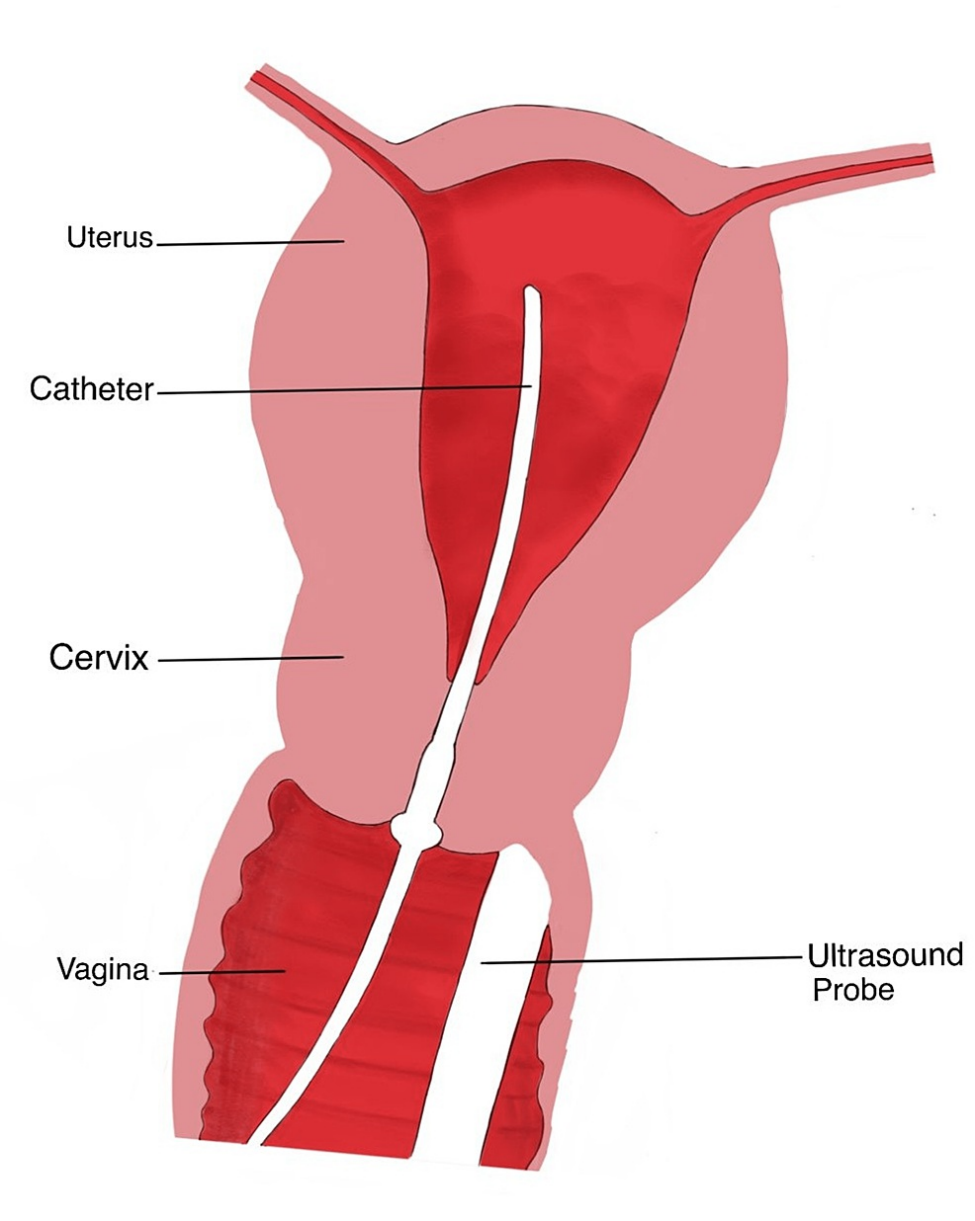
Imaging technique in saline infusion sonohysterography.

However, pre-procedural baseline ultrasound imaging cannot be skipped. Screening of pathologies like hydrosalpinx can be helped by ultrasound, as it can be mistaken for the injected fluid during SIS. Screening of active pelvic inflammatory disease, which is absolutely contraindicated for SIS, is possible by pre-procedural baseline imaging. Determining the size and position of the uterus and cervix for better estimation of the quantity of saline required and better positioning of catheter is aided by pre-procedural imaging [5]. The imaging technique is depicted in Figure 2.

Drawbacks of SIS in diagnosis

The apparent prerequisite for SIS is adequate distension of the uterine cavity; anechoic saline is used for this purpose. Several patient-related and technique-related problems may arise. The most common problem is the inability to access the cervix [5]. This can be avoided by proper positioning of the patient and suitable speculum selection according to the vaginal size. The issue of variable cervical orientation can be dealt with by asking the patient to bear down after maneuvering the speculum to the known position of the external os or using a tenaculum to apply traction on the cervix [5,11]. Cervical dilators or guide wires can be used to push the catheter beyond a stenosed cervix. Non-steroidal anti-inflammatory drugs (NSAIDs) and local anesthetic lubricant jellies can be applied to the instruments to reduce pain.

Shadowing of the concerned pathology to be visualized may be due to air trapped in the uterine cavity. Visualization may be interfered mechanically because of the presence of blood clots within the endometrial cavity, especially in a patient with irregular menstrual cycles as it is challenging to schedule the procedure at the correct time. A blood clot may be mistaken for an endometrial polyp or other focal endometrial lesions. Blood clots can be differentiated from endometrial polyps by doppler imaging which shows an absence of blood flow. Adequate inflation of the balloon is essential in order to prevent dislodgement of catheter [5]. However, over-distension should be avoided to prevent obscuration of the pathology. Another drawback is that the procedure involves uterine distention and may be painful for some patients. The procedure may also introduce infection into the uterine cavity if appropriate aseptic precautions are not taken.

Complications of SIS

Saline infusion sonohysterography, as a procedure has minimal invasion, is comparatively safe and well tolerated by patients, with the number of reported complications being very few. Dessole et al. performed a prospective study that described complications in approximately 8.8% of 1153 patients undergoing SIS [7]. Less than 0.2% of patients reported post-procedural infection [7].

Diagnostic performance of SIS in various uterine pathologies

Submucosal Fibroid

These are benign uterine tumors of fibrous tissue and smooth muscles and are classified as submucosal, intramural, and serosal based on their location. Often asymptomatic, submucous fibroids are incidental findings on imaging; however, symptomatic fibroids are the most common indication for hysterectomy [12,13]. Pelvic pain, AUB, recurrent pregnancy loss, and infertility are the common presentations in symptomatic fibroids [9,10].

Fibroids appear as hypoechoic, round, and broad-based lesions on imaging, whereas endometrial polyps are seen as hyperechoic masses compared to the myometrium. On SIS, loss of endometrial-myometrial interface is seen in fibroids and they subtend an obtuse angle with the endometrium. In contrast, the endometrial-myometrial interface is intact in endometrial polyps and has an acute angle with the endometrium [6]. Although found in less than half the cases, a single feeding artery remains a pathognomic finding for endometrial polyps [9,14,15], whereas uterine fibroids are supplied by a branching vascular pattern [16]. Typically, on ultrasound, uterine fibroids show refractive posterior shadowing [16].

Both surgical and hormone-based treatments are available. Conservative management is usually carried out in a majority of cases, but submucosal fibroids presenting with AUB may be surgically removed. Saline infusion sonohysterography can be used to determine the number and site and volume of projection into cavity of fibroids [16]. Distorted or obscured endometrium may be seen on pelvic ultrasound in patients with multiple fibroids.

Endometrial Polyp

It is a localized, benign proliferation of the endometrial glands and stromal tissue that projects into the endometrial cavity. Varying with population and the diagnostic method used, the prevalence of endometrial polyps is between 16% and 34% [17]. Endometrial polyp is an important etiology for AUB in pre-menopausal and post-menopausal women. In some cases, there may be a co-existence of diffuse endometrial hyperplasia and polyps [18]. Transformation to malignancy is rare; the prevalence is less than 5% [19].

On ultrasound, it appears as a homogenously echogenic polypoid lesion that may undergo hemorrhage, necrosis, and infarction [9,16]. On TVS, it is difficult to differentiate endometrial polyp and diffuse endometrial hyperplasia as both appear as non-specific thickening of echogenic endometrium. In SIS, the endometrial walls are separated by anechoic saline and the lesion is better outlined, which helps better characterize the endometrial lesion. In most cases, an endometrial polyp has a stalk with a single feeding vessel [17]. Echogenic lesions with a smooth outline can also be differentiated from malignancy. Endometrial polyps must be surgically removed, as imaging cannot classify them as benign or malignant [6].

Endometrial Hyperplasia

It is the abnormal diffuse proliferation of stroma and endometrial glands in contrast to focal proliferation seen in endometrial polyps. Depending on the number, presence, and degree of atypia, it is of three types - simple, complex, and atypical hyperplasia.

On SIS, endometrial hyperplasia appears as homogenous endometrial thickening. The endometrial thickening may be heterogenous with cystic degeneration. Focal lesions like fibroids, polyps, or carcinoma may be mimicked by asymmetrical endometrial thickening. The primary treatment options for endometrial hyperplasia, with or without atypia are cyclic progesterone therapy or hysterectomy, respectively [20].

Endometrial Carcinoma

Post-menopausal women are most commonly affected by endometrial carcinoma and account for 10% of cases of post-menopausal bleeding [15]. Unopposed estrogens, such as polycystic ovary syndrome (PCOS), nulliparity, early menarche, late menopause, obesity, and metabolic syndrome, are the major risk factors associated with endometrial carcinoma.

On ultrasound, it appears as polypoid mass, focal endometrial thickening or heterogeneous diffuse endometrial thickening, and disrupted endometrial-myometrial interface. On imaging alone, an early stage of endometrial cancer and endometrial hyperplasia may not be differentiated from each other, and to confirm the diagnosis, histopathology becomes essential. Heterogenous echotexture, distorted endometrial-myometrial interface, fluid in the endometrial cavity, and irregular margins of endometrium point toward a malignancy [9,15]. As opposed to these findings, homogenous echotexture, well-defined endometrial-myometrial interface, cystic changes, and regular margins of endometrium point toward a benign pathology. The mainstay surgical treatment is total abdominal hysterectomy along with bilateral salpingo-oophorectomy. Chemotherapy and radiotherapy may also be used when indicated [21].

Uterine Synechiae

Uterine synechiae, also known as uterine adhesions, is one of the important causes of recurrent abortions and infertility. On routine TVS, these adhesions are difficult to visualize as the endometrium often appears normal [22,23]. Saline infusion sonohysterography provides better visualization of the echogenic bands by separating the uterine walls. Real-time cine imaging offers the best visuals of uterine adhesions, which are seen as irregular echogenic bands present along the distorted endometrial cavity. In advanced stages of uterine synechiae, adequate distension of the uterine cavity becomes difficult. Poor endometrial cavity distension is indicative of uterine synechiae. Endometrial synechiae or adhesions may limit the distension of the endometrial cavity from previous procedures or uterine instrumentation, or infections may lead to tethering of endometrial mucosa with each other. The mainstay of treatment is hysteroscopic guided adhesiolysis. Readhesion may be prevented by the insertion of an intrauterine device [22,23].

Adenomyosis

It is a benign endometrial pathology with the presence of ectopic stroma and endometrial glands in the myometrium with smooth muscle hypertrophy [24]. It may be asymptomatic or present with pelvic pain, AUB, or dysmenorrhea [25]. On sonography, it appears as asymmetrical thickening of the myometrial wall, with globular uterine enlargement, heterogeneous myometrial echotexture, enlarged junctional complex, myometrial cysts, echogenic linear striations, and filling of the myometrial cracks with the fluid in the endometrial cavity [25,26]. The first imaging modality to be used often is TVS, but the diagnosis may not be specific. Compared to ultrasound, features in MRI are more specific. Saline infusion sonohysterography also overcomes the drawbacks of TVS and allows precise localization of sub-endometrial cystic changes [25]. Moreover, better visualization of the endometrium is enabled by distension of the uterine cavity by saline, which can be obscured by the underlying adenomyosis in other imaging modalities. Other non-surgical treatment modalities include endometrial ablative therapy, hormonal therapy, and uterine artery embolization [27].

Congenital Uterine Anomalies

Congenital uterine abnormalities such as bicornuate or septate uterus can be better characterized by SIS. Precise imaging of the septum, its thickness, and type is possible because of uterine cavity distension with saline. Differentiating a septate from a bicornuate uterus is also possible using SIS. Post-operative evaluation of the congenital uterine abnormalities to look for adequacy of repair can also be carried out by SIS.

Tamoxifen Therapy

Tamoxifen is a drug having anti-estrogenic properties. The anti-estrogenic effect of tamoxifen on the breast is used to treat breast cancer. There is a higher incidence of endometrial diseases, such as endometrial cysts, polyps, hyperplasia, and malignancies. On imaging, endometrial hyperplasia accompanied by cystic changes is the most common finding [10]. To monitor endometrial pathologies developing secondary to tamoxifen therapy, SIS acts as a useful diagnostic modality. It has a higher specificity than TVS in assessing pathologies of endometrium in post-menopausal women with breast cancer on tamoxifen therapy who are asymptomatic [28].

Comparison of SIS with other imaging modalities

SIS Versus TVS

In the assessment of the uterus and adnexa, TVS is the mainstay. However, it is limited in finding small intrauterine cavity lesions and differentiating between diffuse and focal endometrial lesions. Different menstrual cycle phases pose a challenge in precise diagnoses of endometrial distortions due to uterine masses. The normal endometrial lining may sometimes be mistaken for endometrial hyperplasia on TVS due to the layers being in close proximity to each other. In contrast to TVS, SIS can be used for imaging focal as well as diffuse endometrial lesions because it enables the assessment of a single layer of endometrial lining [2].

SIS Versus HSG

Infertility is evaluated by tests for tubal factors and patency of the fallopian tube. At present, the screening image modality of choice for tubal patency is HSG [29]. SIS is an efficacious and safe substitute for tubal patency assessment. The advantages of SIS over HSG are its cost-effectiveness, ease of use, absence of ionizing radiation, and ability to visualize the entire uterine cavity concurrently. Disadvantages of SIS include its inability to visualize the whole length of the fallopian tube and confident determination of patency of one or both fallopian tubes [3,4]. Various studies by Christianson et al., Socolov et al., and Campbell et al. showed that SIS is at par with HSG for identifying uterine lesions, the only downside being lesser sensitivity in identifying tubal patency [4,30-32].

SIS Versus Hysteroscopy

Hysteroscopy is the reference standard in the evaluation of lesions of uterine cavity. However, its disadvantages are that it is invasive and expensive and can as well lead to complications, such as bleeding and perforation, because of its invasive nature [33]. The role played by hysteroscopy in evaluating adnexal and myometrial lesions is limited.

Studies by Scwarzler et al. and Epstein et al. have shown comparable specificity and sensitivity of hysteroscopy and SIS in the evaluation of intrauterine lesions and focal endometrial lesions. Although hysteroscopy was superior in differentiating between benign and malignant endometrial lesions, none of them were reliable [34,35].

Therefore, SIS being a non-invasive and cost-effective imaging modality with sensitivity comparable to that of diagnostic hysteroscopy can be utilized as a substitute for initial screening in workup of females with AUB. An upside of hysteroscopy is its ability to perform biopsy concurrently at the time of imaging for histopathological diagnosis which is not possible in SIS.

SIS Versus Pelvic MRI

The use of MRI for evaluating female pelvis is rising because of its better soft tissue characterization, superior contrast resolution, and multiplanar imaging capabilities. MRI is a superior modality of imaging for fibroids. MRI has a larger field of view and is also used in the mapping of adenomyosis pre-operatively [36].

After uterine artery embolization is performed, MRI provides a road map and detects the signal intensity and volume changes of fibroids [37]. MRI is also better in staging and evaluating endometrial cancer because it can determine the pre-operative staging and assess the extent of myometrial invasion [38]. Its ability to demonstrate the widened junctional zone makes pelvic MRI a far more precise modality in assessing adenomyosis [39]. In assessing congenital mullerian duct anomalies MRI is the reference standard [40,41]. The advantages of SIS are its concurrent tubal patency evaluation and assessment of tubal factors of infertility. In patients who are unable to undergo MRI due to contraindications, such as renal failure, metallic implants, and claustrophobia, SIS can be performed. The comparison of SIS with other imaging modalities used in gynecology has been summarized in Table 1.

**Table 1:** Summary of comparison of SIS with other imaging modalities. SIS: sonohysterosalpingography

SIS versus TVS	
SIS	TVS
Better differentiation between diffuse and focal endometrial lesions. Single layer evaluation uterus possible.	Inferior with respect to precision.
SIS versus HSG	
SIS	HSG
Safe, efficacious, cost-effective, and no radiation exposure.	Better and complete fallopian tube visualization.
SIS versus hysteroscopy	
SIS	Hysteroscopy
Safe, non-invasive, non-expensive, and lesser complications. May be used for screening.	Although both are reliable, hysteroscopy is slightly better in differentiating between benign and malignant lesions.
SIS versus pelvic MRI	
SIS	Pelvic MRI
Inexpensive, it can be used in patients with metallic implants.	Better visualization in multiple planes, superior resolution, and larger field of view.

## Conclusions

SIS provides superior visuals of the endometrial pathologies compared to some of the routinely performed imaging modalities in gynecology and helps differentiate them from sub-endometrial causes thus providing superior diagnostic results. It has the additional advantage of concurrent assessment of tubal patency with comparable precision. However, while investigating a case of infertility HSG is the screening modality of choice. Its ability to visualize the complete length of fallopian tube gives it an edge over SIS. In cases related to infertility and abnormal uterine bleeding, SIS plays a big role in diagnostic workup. The drawbacks of SIS owe to the procedure being a bit painful and may lead to the introduction of infection if adequate aseptic measures are not undertaken. SIS can be effectively utilized as an additive screening modality before pelvic imaging. Above all, its cost efficacy and comparable accuracy give it an edge over other imaging modalities where lesions are not clearly visualized. In a country like India which has a large rural population suffering from a plethora of gynecological diseases, SIS being cheap yet effective can be used to screen as well as diagnose some of the diseases.
